# siRNA Therapeutics for the Treatment of Hereditary Diseases and Other Conditions: A Review

**DOI:** 10.3390/ijms26178651

**Published:** 2025-09-05

**Authors:** Alexei Shevelev, Natalia Pozdniakova, Evgenii Generalov, Olga Tarasova

**Affiliations:** 1 Laboratory of DNA metylom and transcriptom editing, Vavilov Institute of General Genetics of Russian Academy of Sciences, Gubkina 3, 119991 Moscow, Russia; shevelab@vigg.ru; 2 Laboratory of radionuclide and radiation technologies in experimental oncology, Blokhin National Medical Research Center of Oncology, Ministry of Health of the Russian Federation, Kashirskoe Shosse 24, 115522 Moscow, Russia; natpo2002@mail.ru; 3 Department of Biophysics, Faculty of Physics, Lomonosov Moscow State University, 119991 Moscow, Russia; 4 Laboratory of Big Data Analysis for Digital Pharmacology, Department of Bioinformatics, Institute of Biomedical Chemistry of Russian Academy of Sciences, Pogodinskaya 10, 8, 119121 Moscow, Russia; olga.a.tarasova@gmail.com

**Keywords:** RNA interference, siRNA, RNA therapy, LNPs, GalNAc conjugates, gene therapy

## Abstract

RNA-based drugs hold significant potential, offering promising new treatments for a wide range of diseases, especially those with a genetic basis. By leveraging RNA interference (RNAi) and other RNA-mediated mechanisms, these therapies can precisely modulate gene expression and address the root causes of genetic defects. RNA-based drugs hold significant potential for treating a range of diseases. However, the transition of these therapies from laboratory research to clinical applications has encountered hurdles. This review explores the composition and outcomes of clinical trials for various modified short RNA drugs. We detail their mechanisms of action, delivery systems—with a focus on lipid nanoparticles and N-acetylgalactosamine (GalNAc) conjugates—and clinical efficacy in treating conditions such as transthyretin (TTR) amyloidosis. Our analysis reveals that while several RNAi-based drugs have achieved clinical approval, a critical unmet need remains: advanced delivery systems capable of precisely targeting diverse tissues, particularly outside the liver. We also underscore the importance of rigorous target validation utilising sophisticated bioinformatics tools and in vitro/in vivo assays to minimise off-target effects and ensure robust therapeutic efficacy. This review proposes a novel framework for optimising RNA drug development, emphasising the crucial interplay between delivery strategies, target specificity, and understanding disease-specific target biology.

## 1. Introduction

Gene therapy is commonly employed to treat recessive hereditary diseases caused by the absence of a functional gene copy. Adeno-associated virus (AAV) capsids are frequently used for this purpose, which highlights the necessity to carefully study the viral immunogenicity, invasion mechanisms, and virus–host interactions in general. For example, Palmieri et al. [1] described a dual-AAV approach that utilises split-intein-based protein trans-splicing for Duchenne muscular dystrophy therapy, addressing the challenge posed by the large size of the dystrophin gene, which is difficult to package within a single viral capsid. Zhang et al. [2] provided an exhaustive review of the use of AAV for the treatment of various forms of hereditary deafness. Non-viral delivery approaches have been developed that allow the introduction of a full-length dystrophin gene into human myotubes [3]. However, all these methods are unsuitable for the therapy of genetic defects of a dominant nature because of the expression of defective copies of genes with abnormal function. For example, Vulto–van Silfhout–de Vries syndrome is caused by de novo variants of the DEAF1 gene [4].

**Figure 1:**
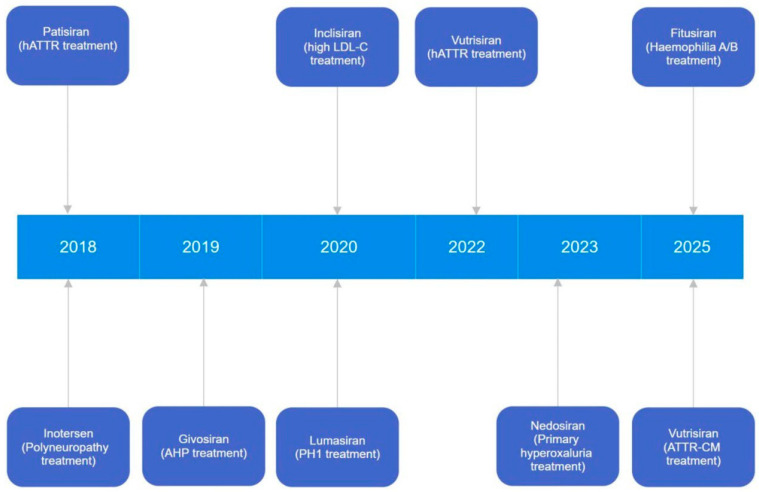
Timeline of siRNA drug approval.

RNA interference (RNAi)-based methods are currently actively used for the therapy of such diseases since the very discovery of the RNAi phenomenon in 1998 [5]. Registration of the first RNAi drug took approximately 20 years (Figure 1) after the discovery of RNAi [6]. These years focused on the development and delivery of small interfering RNAs (siRNAs) to target cells, aiming to reduce the expression of abnormally functioning genes.

We provide an overview of candidate RNA-based drugs that have undergone clinical trials as therapeutics for inherited diseases, as well as remedies for vascular ischaemia and wound healing.

## 2. RNA Therapeutics

### 2.1. Patisiran—A First-Generation Therapeutic for Patients with Hereditary Transthyretin-Mediated Amyloidosis (hATTR)

The first drug based on the principle of RNAi, patisiran, developed by Alnylam Pharmaceuticals for the treatment of amyloid polyneuropathy, was approved for use in the United States in 2018. The drug is a double-stranded RNA with a duplex length of 19 nt and two protruding nucleotides on each side of the 3′-ends (the total length of each chain is 21 nt) [7]. The drug is designed to inhibit the overproduction of an abnormal transthyretin (TTR) protein, a membrane transporter responsible for carrying thyroxine and retinol. This abnormal form arises from a mutation in the TTR gene, which impairs its ability to form stable tetramers. Consequently, this disruption leads to the production of proteolysis-resistant amyloid within the cytoplasm. Amyloid accumulation can occur in different tissues, but this process leads to the most severe consequences for the patient in the case of brain and myocardium damage. The siRNA included in patisiran does not distinguish between normal and rearranged forms of TTR.

Significantly, 2-methyl-O-uridine is used instead of uridine as part of the sense chain; the end protruding beyond the duplex is formed by deoxythymidine, and cytidine is replaced by 2-methyl-O-cytidine [8]. The composition of the antisense chain has fewer modified nucleotides: only two uridine residues out of seven are substituted for 2-methyl-O-uridine; in addition, the 3′-end of the antisense chain protruding outside the duplex, as in the case of the sense chain, is formed by two deoxythymidine residues. The choice of this particular order of using modified nucleotides is due to the need to achieve a compromise between increasing the resistance of synthetic RNA to degradation by nucleases during circulation in the blood and, on the other hand, preserving the ability of the duplex to trigger RNA-induced silencing complex (RISC). At the same time, patisiran may penetrate into neurons, where the destruction of defective TTR messenger RNA (mRNA) occurs [7]. This leads to an improvement in neuropathy scores, indicating a decrease in the manifestation of neurodegeneration symptoms.

**Figure 2:**
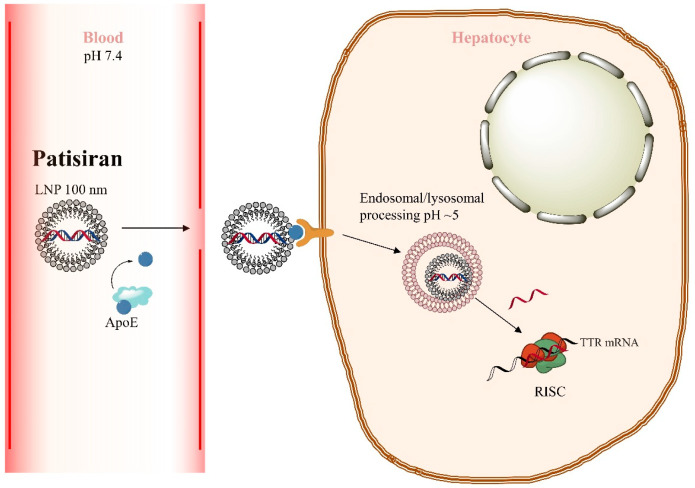
The pharmacokinetics of patisiran. Pharmacokinetics of patisiran following intravenous injection: sequence of cellular events. PEG2000-C-DMG coating dissociates from the LNP. After that, endogenous ApoE binds with the LNP, activating ApoE-dependent endocytosis by hepatocytes. Upon internalisation, the ionisable lipid DLin-MC3-DMA is protonated, subsequently interacting with the negatively charged lipids of the endosome. This interaction disrupts the integrity of both the LNP and the endosomal membrane, facilitating the release of ALN-18328 into the cytoplasm. ALN-18328 binds to RISC, which degrades TTR mRNA [8]. Arrows depict the uptake and intracellular trafficking of patisiran, leading to its assembly within the RISC complex.

Patisiran’s drug formulation employs second-generation cholesterol-containing lipid nanoparticles (LNPs) (Figure 2), along with the polar lipid 1,2-Distearoyl-sn-glycero-3-phosphocholine (DSPC), the 1,2-Dimyristoyl-sn-glycero-3-methoxypolyethylene glycol (PEG2000-C-DMG), and the ionisable aminolipid DLin-MC3 DMA as the vehicle for siRNA delivery [9] Cholesterol and DSPC are thought to increase drug stability during storage, PEG2000-C-DMG increases stability during circulation in the blood, and DLin-MC3-DMA promotes LNP uptake and release by target cells. Zhang et al. [8] reviewed the pharmacokinetics of patisiran in detail. Based on their observations, the intravenous administration of the drug initiates a cascade of events. After intravenous injection of patisiran, the PEG2000-C-DMG component dissociates from the complex. The removal of the PEG2000-C-DMG moiety coating enables the association of endogenous apolipoprotein E (ApoE) with the LNP, facilitating uptake into hepatocytes through an ApoE-dependent mechanism. Upon internalisation via endocytosis, the ionisable lipid DLin-MC3-DMA becomes protonated (positively charged) due to the decreasing pH within the endosome. The positively charged DLin-MC3-DMA then interacts with the negatively charged endosomal lipids, leading to disintegration of the LNP, destabilisation of the endosomal membrane, and release of ALN-18328 into the cytoplasm. The liberated ALN-18328 binds to the RNA-induced silencing complex (RISC), resulting in the targeted degradation of TTR mRNA and a subsequent reduction in TTR protein levels. Finally, a portion of the internalised LNPs undergoes exocytosis from late endosomes/lysosomes, returning to the circulation.

Unfortunately, the large size of the LNP (about 100 nm) makes patisiran insufficiently stable during storage and difficult to administer. Its administration is possible only in hospital settings [7]. Given the complexity and unsafety of use [10], it can be expected that patisiran will soon be displaced from the market by the more advanced drugs inotersen and vutrisiran.

### 2.2. Vutrisiran—Second-Generation Therapeutic for Patients with hATTR

**Figure 3:**
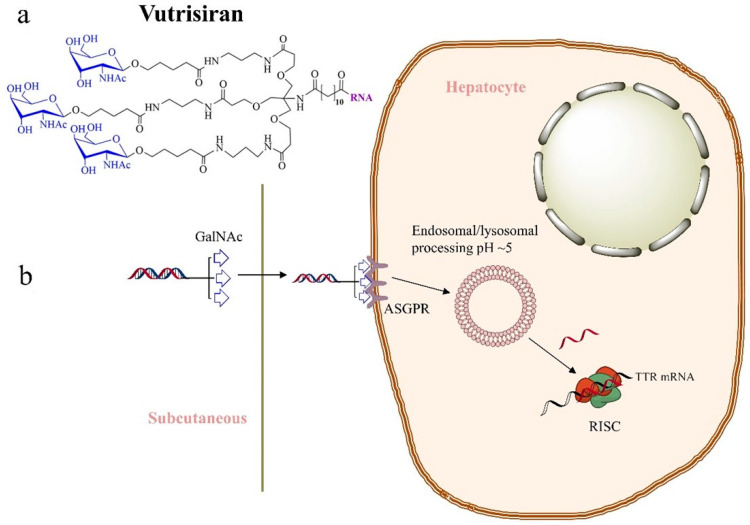
Vutrisiran mechanism of gene silencing. (**a**) Structure and (**b**) pharmacokinetics of vutrisiran. Arrows depict the uptake and intracellular trafficking of vutrisiran, leading to its assembly within the RISC complex.

Vutrisiran, a development by Alnylam Pharmaceuticals to replace patisiran, was approved for use in the U.S. and EU in 2022. Vutrisiran is a double-stranded RNA (dsRNA) with one chain of 21 nt and another of 23 nt. Both strands are fully modified: 35 nucleotides contain a 2′-O-methyl (2′OMe) substitution, and 9 contain a 2′-fluoro (2′-F) substitution [11]. The duplex contains a total of six phosphorothioate (PS) bonds located near the ends of both chains. It is conjugated to N-acetylgalactosamine (GalNAc) to ensure delivery of dsRNA to target cells (Figure 3). Vutrisiran specifically targets the TTR mRNA in liver cells. Once inside, vutrisiran integrates into the RISC, guiding it to cleave and destroy the TTR mRNA, reducing the liver’s ability to synthesise both normal and variant TTR proteins that contribute to amyloid formation. By substantially decreasing TTR production, vutrisiran aims to slow down or halt the progression of ATTRv amyloidosis, thus alleviating the debilitating symptoms associated with the deposition of amyloid fibrils throughout the body. Conjugation with GalNAc provides highly efficient targeted delivery to hepatocytes, which are the main site of TTR production [12]. As with patisiran, the siRNA incorporated into vutrisiran does not distinguish between normal and rearranged forms of TTR.

In clinical trials, vutrisiran showed a significantly lower rate of undesirable side effects compared to patisiran [13], although its administration was performed subcutaneously without following the complex preparatory procedure of dexamethasone premedication required in the case of severe allergic or inflammatory reactions [14].

### 2.3. Givosiran/Givlaari—A Second-Generation Therapeutic for Patients with Acute Hepatic Porphyria

In 2019, the FDA approved givosiran (Alnylam Pharmaceuticals, Cambridge, MA, United States), which treats acute hepatic porphyria (AHP), for use in the United States. AHP is an orphan disease [15], resulting from a mutation in one of the genes of the heme synthesis pathway. Mutations disrupt feedback control in the heme synthesis pathway, leading to δ-aminolevulinate synthase 1 (ALAS1) hyperproduction [16]. Overactivity of ALAS1 then causes the production of neurotoxic metabolites, aminolevulinic acid, and porphobilinogen. The accumulation of these metabolites results in a dramatic decrease in quality of life, marked by severe abdominal pain, vomiting, hypertension, tachycardia, altered mental status, seizures, and muscle weakness [17]. In some cases, AHP patients develop paralysis and kidney and liver dysfunction [18].

Givosiran (ALN-AS1) is a double-stranded GalNAc-conjugated siRNA drug designed to specifically recognise ALAS1 mRNA. Specifically, givosiran targets the region between nucleotides 918 and 937 of the ALAS1 mRNA. During clinical trials, givosiran provided a 74% reduction in the frequency of the main symptoms of AHP [19]. However, long-term use of this drug caused decreased renal function and liver failure [20,21].

### 2.4. Lumasiran/Oxlumo—A Second-Generation Therapeutic for Patients with Primary Hyperoxaluria Type 1 Targeting Glyoxylate Oxidase

Lumasiran (Alnylam Pharmaceuticals), which treats primary hyperoxaluria type I (PH1), was approved for use in the EU and the U.S. in 2020. This drug treats PH1, a congenital orphan disease with rapidly progressive urolithiasis as a symptom. Lumasiran has as its molecular target the mRNA of the enzyme glyoxylate oxidase (GO), the overproduction of which in the liver is ultimately responsible for excessive accumulation of oxalate in the urine. In lumasiran, the siRNA traditionally consists of a 23-base guide strand and a 21-base passenger strand. It is fully modified with 10 2′-F-substituted nucleotides and the other 34 2′-Ome-substituted nucleotides [22]. Researchers used covalent modification of the GalNAc sense chain for delivery [23]. In trials, lumasiran dosing caused a 75% mean maximal reduction in urinary oxalate (UOx) levels. Patients who received 3 mg/kg of lumasiran monthly or quarterly were able to achieve normal UOx levels. The side effects recorded during the three phases of clinical trials were found to be insignificant [6].

### 2.5. Nedosiran—A Second-Generation Therapeutic for Patients with Primary Hyperoxaluria Type 1 Targeting Lactate Dehydrogenase

In 2023, a competitor of lumasiran—nedosiran (Rivfloza), a drug for the therapy of primary hyperoxaluria—was approved for use in the United States [24]. Since the target of nedosiran, unlike lumasiran, is the mRNA of lactate dehydrogenase (LDH), the final enzyme responsible for oxalate synthesis in the liver, it can exert therapeutic effects in all types of hyperoxaluria, not just type 1 [23].

As with nedosiran, it utilises Dicerna Pharmaceuticals’ proprietary methodology for delivering RNA to the liver by conjugating the sense chain to GalNAc-siRNA, similar to Alnylam’s ESC technology. The active ingredient molecule of nedosiran consists of a 22 nt long antisense chain and a 36 nt long sense chain, which form a tetracyclic configuration. Almost all the bases are modified: 19 of them have a 2′-F ribose residue modification, and 35 have a 2′-OMe modification. Six main chain bonds near the ends of the duplex are replaced by PS groups. Conjugation of RNA with GalNAc provided good absorption of the drug by the liver after subcutaneous administration [25].

During Phase 1 clinical trials, it was found that 24 h after nedosiran administration, the level of oxalate in urine decreased by 24.5%, whereas in the placebo group it tended to increase. In three out of four patients receiving nedosiran, after the first use, there was an observed decrease in UOx within 24 h of more than 30%, and one level approached normal [24].

### 2.6. Inclisiran/Leqvio—A Second-Generation Therapeutic for Adults with Primary Hypercholesterolaemia or Mixed Dyslipidaemia

Inclisiran (Novartis, Basel, Switzerland) received approval for the treatment of hypercholesterolaemia in the EU in 2020 and in the U.S. in 2021. This RNAi therapeutic inhibits the synthesis of proprotein convertase subtilisin/kexin type 9 (PCSK9) [26]. A well-studied function of PCSK9 is to bind the EGF-A domain of the low-density lipoprotein receptor, resulting in degradation of the receptor. Reduction in LDL receptor levels on the cell surface reduces cellular uptake of low-density lipoprotein, which may lead to hypercholesterolaemia. Thus, suppression of PCSK9 expression through the action of inclisiran can reduce high-density cholesterol in the blood. This fact makes inclisiran a more effective substitute for traditional drugs to reduce the hyperlipidaemia class statins [27]. In addition, PCSK9 plays a role in the differentiation of cortical neurones, which allows us to expect neurogenic effects from the drug inclisiran [28]. During preclinical trials of inclisiran in monkeys, an observed reduction in plasma PCSK9 levels by more than 80% and in low-density lipoprotein cholesterol (LDL-C) by about 60%. This effect was maintained for more than 30 days. Key metabolite levels returned to baseline levels only 90–120 days after administration of the drug [26].

Inclisiran is structurally similar to givosiran in terms of its active substance, differing only by the presence of a 2′-O-methoxyethylribose (2′-MOE) substituent in the ribose ring of one of the nucleotides. It consists of a 23-base guide strand and a 21-base passenger strand. Inclisiran is fully modified with one 2′-MOE, 11 2′-F, and 32 2′-OMe substitutions. Six PS modifications are distributed across the termini of the strands, and GalNAc conjugation allows for high hepatocyte uptake after subcutaneous administration [29]. A Phase 2 clinical trial of inclisiran called ORION-2 (NCT02963311) enrolled 501 patients with elevated serum LDL-C levels and high cardiovascular (CV) risk who were already receiving the maximum tolerated dose of statins and/or ezetimibe [30]. Inclisiran has clear advantages in terms of duration of action and production cost over current drugs based on humanised antibodies against PCSK9: alirocumab and evolocumab (Repatha) [31]. At the same time, according to [32], the reduction in LDL-C in patients’ blood found in the ORION-9, ORION-10, and ORION-11 trials was insignificant—only a 2.5% reduction in the risk of major CV damage compared with placebo, which casts doubt on the utility of its use.

### 2.7. Fitusiran—A Second-Generation Therapeutic for Patients with Haemophilia A and Haemophilia B

Fitusiran is intended to treat haemophilia. Haemophilia A and B are X-linked clotting disorders caused by a deficiency of clotting factors VIII and IX, respectively [33]. The current standard of care for haemophilia consists of frequent (2–3 times a week) prophylactic infusions of concentrated clotting factor preparations to replenish the spent factor [34]. This regimen is costly, invasive, and burdensome. By degrading the mRNA of antithrombin, an endogenous anticoagulant, fitusiran increases the activity of thrombin, an enzyme that promotes blood clotting, thereby normalising haemostasis. This mechanism may be effective regardless of the presence or absence of inhibitory antibodies.

Like inclisiran and givosiran, fitusiran utilises the principle of RNA conjugation with GalNAc, patented by Alnylam. As in the case of other drugs developed by Alnylam, RNA in fitusiran is fully modified by ribose atoms: it contains 21 2′-F and 23 2′-OMe modifications. There are 6 PS-type bonds located near the ends of the RNA duplex [35]. The covalent attachment of triGalNAc to the sense chain of RNA provides targeting of the active substance to the liver, where antithrombin production occurs. Although Phase 1 and 2 clinical trials showed encouraging results, with improved blood clotting efficiency in the majority of patients, the trials of this drug were suspended in 2017. Currently, fitusiran is not authorised for use in any country in the world.

### 2.8. Teprasiran—A p53-Repressing Therapeutic for Treatment of Delayed Graft Function (DGF)

Teprasiran, developed by Quark Pharmaceuticals and transferred for further commercialisation to Novartis, is an agent to reduce the level of expression of the known anti-oncogene p53 [36]. The target of teprasiran is acute kidney injury (AKI), which often develops in kidney transplant patients (delayed graft function (DGF)) and patients who have undergone cardiac surgery [37]. Unlike most previously discussed siRNA-based therapeutics, teprasiran is only minimally modified. It consists of two 19 nt chains, each with 19 2′-OMe ribose modifications. The sugar–phosphate backbone remains unmodified. Notably, teprasiran is administered as a free miRNA molecule without a delivery system or conjugated targeting ligand. As previously discussed, systemically administered free siRNA is largely absorbed and excreted by the kidneys. When teprasiran is used to treat DGF, this type of biodistribution is desirable. When administered intravenously, teprasiran in its pure form is rapidly excreted by the kidneys, then reabsorbed by proximal tubule cells (PTC) where it accumulates [38]. Despite the lack of modifications and targeting ligands, teprasiran achieves significant cellular internalisation due to the special anatomy of PTC. miRNA binds intensively to the brush border of proximal tubules and subsequently undergoes endocytosis [6]. Thielmann et al. [39] reported the results of the teprasiran clinical trial. A total of 360 patients were evaluated. No significant differences were observed in the overall population with respect to major adverse renal events at day 90 of treatment. Despite the outcome of the trials, teprasiran has not been authorised for use in any country in the world to date.

### 2.9. Cosdosiran—A Therapeutic for Patients with Nonarteritic Anterior Ischaemic Optic Neuropathy and Primary Angle Closure Glaucoma

Cosdosiran is another development by Quark Pharmaceuticals that did not involve Alnylam. It is intended for the therapy of eye diseases—nonarteritic anterior ischaemic optic neuropathy (NAION) and primary angle closure glaucoma. NAION and glaucoma are both ocular diseases characterised by loss of retinal ganglion cells (RGCs) as a result of apoptosis [40]. Because RGCs are unable to divide and replenish their pool, their gradual loss eventually leads to visual impairment and blindness. Cosdosiran induces the destruction of caspase-2 mRNA, a protein that plays a key role in triggering apoptosis in RGCs [41]. Like teprasiran, cosdosiran contains dsRNA consisting of two chains of 19 nt in length, each with nine modifications of the 2′-OMe ribose residue in the guide chain and L-deoxycytidine at the ends of the sense chain [42]. Cosdosiran RNA has a natural structure of a sugar–phosphate backbone and has no groupings responsible for the targeted delivery of the active substance. Cosdosiran is administered directly into the eye, eliminating the need for specific preparatory groupings. Combined with the benefits to the ocular immune system, this route of administration allows cosdosiran to circumvent many of the pharmacokinetic barriers associated with systemic administration. Although cosdosiran’s RNA modifications are smaller than those of other siRNA-based agents, they are sufficient to provide action. The molecule does not have any appreciable immunostimulatory activity and remains chemically stable in intraocular fluid [43]. In animal tests, RGCs were found to efficiently uptake cosdosiran siRNA molecules. Such uptake may be due to the prolonged retention of the drug in the vitreous body, which is in direct contact with the retinal ganglion cell layer [42]. The developer completed Phase 1 and 2 clinical trials and initiated Phase 2b/3 trials, but these were halted in 2020 when the sponsor discontinued funding. The FDA has registered cosdosiran as an agent for the therapy of orphan diseases.

### 2.10. Tivanisiran—An siRNA-Derived Therapeutic for the Treatment of Dry Eye Disease

Tivanisiran, developed by Sylentis S.A., is designed to reduce pain in patients with dry eye syndrome (DED). Tivanisiran targets the mRNA of transient receptor potential cation channel subfamily V member 1 (TRPV1), a channel involved in pain signal transduction, fibrogenesis modulation, the stress response, and the innate inflammatory response [44]. TRPV1 has been proven to play a significant role in mediating enhanced nocifensive behaviour in DED [45]. Tivanisiran substance is an siRNA consisting of ribonucleotides without any modification. No delivery vehicles were used in the preparation, as it was assumed that the siRNA would easily reach the target cells by direct injection onto the cornea. The trials showed that the drug was not effective in terms of pain reduction but had an effect at the level of a secondary symptom—reduction in abnormal corneal pigmentation and conjunctival hyperaemia. As a result of the trials, the drug was not approved. However, during the trials, a positive effect was observed in patients with Sjogren’s syndrome, which is characterised by the presence of DED.

### 2.11. Cemdisiran—An siRNA-Derived Therapeutic for Adults with Immunoglobulin A Nephropathy (IgAN)

Cemdisiran, a drug developed by Alnylam Pharmaceuticals, is designed for the therapy of IgA nephropathy (IgAN). It targets the complement component 5 (C5) mRNA. This protein is produced in the liver and further circulates in the blood, thereby facilitating siRNA-based drug delivery. Gaya et al. [46] provided the results of a Phase 1/2 clinical trial of cemdisiran are summarised. The drug was administered weekly at a dosage of 400 or 600 mg. Administration of cemdisiran caused a persistent decrease in the activity of the complement system. According to the trial results, cemdisiran monotherapy was insufficient to prevent haemolysis in patients with paroxysmal nocturnal haemoglobinuria (PNH), as evidenced by serum LDH levels. Combination therapy with cemdisiran and eculizumab reduced the dose of eculizumab required to provide adequate control of intravascular haemolysis. These results demonstrate the potential benefit of concomitant use of cemdisiran in patients who respond inadequately to eculizumab monotherapy. Despite partial success, the cemdisiran trial was completely discontinued in 2024.

### 2.12. MRG110 (Anti-miR-92), for Wound Healing and Preventing Heart Failure (HF)

The MRG110 drug was developed by miRagen Therapeutics (Boulder, CO, USA). The developers do not disclose the sequence of the RNA or the composition of the delivery system. It is only known that the drug is intended for intradermal injection.

Gallant Behm et al. [47] reported that miR-92a, present in vascular endothelial cells, has a strong potential to suppress angiogenesis. In turn, decreasing the expression level of this RNA leads to increased angiogenesis in many organ systems, which theoretically may contribute to the restoration of myocardial function after infarction and skeletal muscles of the limbs after ischaemia and promote vascular repair after injuries of various types. Inhibitors of miR-92a are considered the basis of therapeutic agents to accelerate skin wound healing.

In preliminary experiments on animals, MGR-110 (miR-92a inhibitor) showed an increase in the formation of new blood vessels, improved blood circulation, and a positive effect on heart function. The drug accelerated wound healing and protected against atherosclerosis, narrowing, and impaired permeability of blood vessels. In particular, MRG-110 was studied on sterile wounds in type 2 congenital diabetes (db/db) mice and healthy pigs. In both acute and chronic wounds, MRG-110 accelerated granulation tissue formation and angiogenesis, the rate of wound healing as measured by time to closure and percentage of closure over time. The effects of MRG-110 were significantly stronger than those observed in comparison groups where purified recombinant human VEGF-165 and platelet-derived growth factor (PDGF-BB) proteins were used for wound healing. In addition, MRG-110 was able to enhance the effect of recombinant human PDGF-BB when co-injected into the wounds of db/db mice. MRG-110 was found to enhance the expression of the pro-angiogenic miR-92a target gene integrin αV in vitro in both human vascular endothelial cells and primary human skin fibroblasts and in vivo in mouse skin, demonstrating its targeted effects in vitro and in vivo. The level of toxicological safety of MRG-110 in experiments on mice and pigs was satisfactory. The results of these studies allowed the developers to proceed with the implementation of Phase 1 clinical trials of MRG-110.

Despite promising preclinical data, MRG-110 is not currently registered or authorised for use in any country.

### 2.13. Remlarsen/MRG201 (miR-29 Mimetic), for Prevention of Fibrous and Keloid Scar Formation

Remlarsen/MRG201, developed by miRagen, is intended for the therapy of fibrotic diseases. As with other miRagen developments, the composition of the drug has not been disclosed. Remlarsen/MRG201 contains the miR-29 mimetic with improved chemical stability achieved through modifications to the ribose residue. Further attempts to improve its properties include, for example, the use of various delivery systems. For instance, as a delivery vehicle, chemical conjugation of RNA with a specific bicyclic peptide BiPPB ligand of PDGFβRβ, which is described in [48], has been used. Chioccioli et al. [49] provided the results of the remlarsen/MRG201 trial for pulmonary fibrosis therapy are reported. The ability of MRG-229 (a new miR-29 mimetic conjugated with the BiPPB analogue of MRG201) to inhibit fibrosis development was investigated on TGF-β1-treated human lung fibroblasts (NHLF), human lung biopsy specimens (hPCLS), and with bleomycin in vivo. Toxicologic studies were performed in rats and primates other than humans. Finally, miR-29b levels (associated with increased mortality) were investigated in cohorts of 46 and 213 patients diagnosed with idiopathic pulmonary fibrosis (IPF) formed at Yale and Nottingham Universities, respectively.

The study further demonstrated that low serum or plasma levels of miR-29 may be associated with increased mortality in IPF patients, suggesting its potential utility in identifying individuals who could derive a survival benefit from MRG-229 treatment.

In both models, peptide-conjugated MRG-229 inhibited the expression of profibrotic genes and collagen. In bleomycin-treated mice, MRG-229 suppressed profibrotic signalling pathways. In experiments with rats and monkeys, the miR-29 peptide-conjugated mimetic was well-tolerated at clinically relevant doses without any observed side effects. Decreased miR-29 concentrations in human peripheral blood from IPF patients were associated with increased mortality in two groups potentially identified as the target population for treatment [49]. Despite its claimed high safety and efficacy, MRG201 is not currently authorised for use in any country in the world.

### 2.14. Lademirsen/RG012 (Anti-miR-21), for Treatment of Alport Syndrome

RG-012 is a drug for the treatment of Alport syndrome developed by Regulus Therapeutics (San Diego, CA, USA). The U.S. FDA and the European Commission have identified it as a possible treatment for an orphan disease. The drug is a modified antisense oligonucleotide, but the developer has not disclosed its sequence or the composition of its delivery system in target cells. Gomez et al. [50] provided the composition of several oligonucleotide variants that were tested during the development of RG-012. Publicly available information does not confirm the use of any of these as the final active ingredient tested in clinical trials.

Alport syndrome, a genetic disorder, is caused by mutations or defects in the COL4A3, COL4A4, or COL4A5 collagen genes [51]. Collagen of the relevant types is essential for maintaining the integrity of renal structure. These defects cause scarring of the basal glomerulus membrane (GLM), which is involved in primary filtration in the kidney. Such scarring leads to progressive loss of renal function, eventually requiring the patient to undergo frequent dialysis or kidney transplantation [52].

Clark [53] found that the kidneys of Alport syndrome patients exhibit overexpression of miR-21, which suppresses protein synthesis of the peroxisome proliferator-activated receptor (PPARα) signalling pathway. RG-012 is a small RNA complementary to miR-21.

Preclinical trials in a mouse model of Alport syndrome showed that RG-012 inhibits the synthesis of miR-21. Inhibition slowed the rate of thickening of the glomerular basement membrane. Administration of RG-012 had a positive effect on renal function. Rubel et al. [54] demonstrated that a Col4a3-/- mouse model showed that combined therapy with anti-miR-21 and angiotensin-converting enzyme inhibitor (ACEi) suppressed fibrosis progression, reduced proteinuria, normalised renal function, and improved survival.

In an F1 animal model with an intermediate stage of Alport syndrome progression, separate treatments with anti-miR-21 and ACE inhibitors (ACEi) both improved renal function and survival. However, the combination of anti-miR-21 and ACEi had a significantly greater effect, particularly on survival.

Clinical trials revealed that RG-012 was ineffective and poorly tolerated, leading the FDA and the European Commission to reject its authorisation for clinical use [55,56].

Sahraei et al. [57] reported that when working in mouse models of human tumour xenografts, suppression of miR-21 expression in tumour-associated macrophages suppresses neoangiogenesis in tumours and induces a beneficial immunostimulatory effect to help inhibit tumour growth. The article [58] found that hyperproduction of miR-21 in vascular endothelial cells promotes activation of JNK1 and JNK2 signalling pathways and production of proinflammatory secretory factors IL-6 and TNF-α, which together increase the risk of cerebral arterial aneurysm rupture.

While suppressing miR-21 expression shows promise for treating Alport syndrome, cancer, and CV diseases, the limited efficiency and targeting of current miR-21 inhibitor delivery methods present significant challenges. Although delivery to renal glomerulus cells and vascular endothelium is relatively straightforward, existing tools cannot achieve satisfactory results, and unknown targets of miR-21 and its inhibitors raise concerns about potential side effects. These knowledge gaps regarding the expression profile and molecular target spectrum of miR-21 impede efforts to eliminate the unwanted side effects of lademirsen/RG012 in the near future.

### 2.15. RNA-Based Drugs and Clinical Trials

siRNA-based drug development is attracting considerable interest among researchers worldwide. RNAi technology presents a strong potential for pharmaceutical development because of its rapid development cycle, the relatively low cost and standardised production of oligonucleotides compared to traditional chemicals and protein-based drugs, and the potential for long-lasting therapeutic effects (3–6 months after one or two administrations). In theory, RNAi also offers highly targeted pharmacological action. The RISC complex, which mediates RNAi, preferentially binds to target mRNA with perfect sequence complementarity, and even a single nucleotide mismatch can significantly reduce its effectiveness. Despite these advantages, RNAi-based therapies have not yet reached their full potential in clinical practice.

While several companies are exploring siRNA-based therapeutics, Alnylam Pharmaceuticals has been responsible for the vast majority of significant successes in clinical trials, sometimes acting in collaboration with larger pharmaceutical companies like Novartis, Sanofi, and Dicerna. Quark Pharmaceuticals and Sylentis S.A. had to abandon their attempt to complete clinical trials despite the presence of certain positive results and satisfactory tolerability of the tested drugs. The review [59] recognises the lack of meaningful progress in China in this area. When analysing the developments of Alnylam Pharmaceuticals itself, it becomes clear that after more than 10 years of trials, approvals were obtained for 6 out of 8 candidates, and in two cases, the trial initiators refused to end the trials. At the same time, in most cases, rare diseases were chosen as the object of treatment, rather than socially significant diseases affecting a significant part of the world’s population or a particular country. They were chosen according to the principle of accessibility for delivery. For the most part, these are diseases where the target cells that need suppression of target gene expression are located in the liver. Alternatively, they may be located in the kidneys, in the retina of the eye, or randomly located for each patient (amyloid polyneuropathy).

While the technology of siRNA-based drug production remains in its early stages, significant challenges remain. The difficulty of delivering siRNA to tissues besides the liver, kidney, and vascular endothelium limits the suitability of this approach for treating CV, oncological, infectious, and autoimmune diseases. Further research is needed to fully evaluate the potential of RNAi technology, and a key question is whether the guide RNA and target mRNA must be perfectly matched to initiate self-replication of the RNAi response. So, several articles [60,61] answer this question in the affirmative, whereas [7] suggests that a match of 7 of 22 nts is sufficient to trigger RNAi. These works are based on the analysis of individual cases that came to the attention of one or another experimental group. Meanwhile, a detailed answer to this question is crucial both for the development of biological science in general and for assessing the prospects of RNAi technology in pharmaceuticals.

A brief view of the RNA-based drugs currently authorised for clinical trials is presented in Table 1. The table includes 14 drugs, of which four gained approvals for unrestricted use in the U.S. or the EU. Approved RNA-based therapies include patisiran (Onpattro/ALN-TTR02), givosiran (Givlaari), lumasiran (Oxlumo), and inclisiran (Leqvio).

As for patisiran, givosiran, lumasiran, and inclisiran, they have many common features. Alnylam Pharmaceuticals developed all of these drugs (Novartis commissioned the development of inclisiran). According to Alnylam Pharmaceuticals’ classification, patisiran belongs to generation 1, while givosiran, lumasiran, and inclisiran belong to generation 2. A common mechanism of action for all four drugs is the use of siRNAs targeting specific mRNAs with abnormally high expression levels in the liver that lead to pathology. These transcripts are ALAS1, GO, and PCSK9. In siRNA development, modifications of the hydroxyl 2 ribose of both 2′-F and 2′-Ome chains were used, while inclisiran development also utilised 2′-MOE. To enhance stability and maintain activity within the Ago2/RISC complex, approximately half of the residues were modified, according to the researchers. Patisiran uses lipid nanoparticles ~100 nm in diameter to deliver siRNA. These LNPs consist of cholesterol, DSPC, PEG2000-C-DMG, DLin-MC3, and DMA. Givosiran, lumasiran, and inclisiran, however, employ a different approach: they directly target siRNA to hepatocytes by covalently linking triantennary GalNAc to the 3′-end of the sense strand. This approach is effective because hepatocytes express the Gal/GalNAc receptor system, while other mammalian cell types do not, providing targeted delivery [62]. By using this approach, it was possible to create drugs suitable for subcutaneous administration, whereas patisiran could only be administered by infusion for several hours after dexamethasone premedication. The half-life of siRNA conjugated with triantennary GalNAc is about 6 h, and the complete disappearance of the drug from the bloodstream occurs ~48 h after administration [63]. An analysis of published data indicates that the recommended dosages of first- and second-generation siRNA-mimetic drugs have remained relatively consistent, ranging from 1 to 10 mg per kg of the patient’s weight.

Table 2 contains the comparison of administration route, efficacy, and side effects for the investigated drugs.

Table 2 summarises the efficacy, side effects, and approval dates (if applicable) for various drugs. Approved drugs like patisiran and vutrisiran (both siRNAs) have proven effective in treating conditions such as hATTR amyloidosis. These drugs demonstrated strong and clinically significant efficacy with acceptable safety profiles. In contrast, inotersen, while effective, carries higher safety risks that necessitate careful monitoring. However, developmental failures like teprasiran and cosdosiran highlight that a tolerable safety profile alone is not enough. Both drugs failed to meet their primary efficacy endpoints in Phase 3 trials, resulting in an unfavourable risk–benefit ratio. For example, teprasiran failed to significantly reduce major adverse kidney events after cardiac surgery compared to placebo in its Phase 3 trial, and a higher incidence of hypotension was noted. Similarly, cosdosiran’s Phase 3 trial did not show significant visual improvement over placebo. These examples demonstrate that robust clinical efficacy is crucial for approval, even with a tolerable safety profile.

## 3. Discussion

This review provides an overview of the current landscape of RNA-based therapeutics, their potential, and their challenges. The development of siRNA-based drugs has garnered significant attention owing to their rapid development cycle, potentially lower cost compared to traditional pharmaceuticals, and the prospect of long-lasting therapeutic effects. The mechanism of RNAi, mediated by the RISC, is a promising target for pharmacological action, with the guide strand of the siRNA theoretically binding specifically to target mRNA. However, as this review demonstrates, the development process is complex, with different hurdles to overcome.

The success of drugs like patisiran, givosiran, lumasiran, and inclisiran demonstrates the viability of RNAi as a therapeutic strategy. These drugs share common features: targeting mRNAs with abnormally high expression levels in the liver (ALAS1, GO, and PCSK9), utilising chemically modified siRNAs to enhance stability and minimise off-target effects, and employing either LNPs or GalNAc conjugation for delivery.

However, there are also limitations of RNAi therapeutics. The majority of approved and clinically advanced siRNA-based drugs target diseases where delivery to the liver is relatively straightforward because of its natural uptake mechanisms. This raises the following question: can RNAi therapies be effectively expanded to treat diseases in other tissues and organs? The challenges remain significant for delivering siRNA to tissues beyond the liver (e.g., CV, oncological, and autoimmune).

One of the main flaws of the LNP delivery system is its tendency to rapidly accumulate in the liver. This liver tropism severely limits the amount of therapeutic RNA that can reach other organs. There are several approaches to overcome these limitations. These include the following: exploiting active targeting strategies involving the conjugation of ligands to the LNP surface that bind specifically to receptors upregulated on target cells in the diseased organ, effectively acting as molecular zip codes to guide the nanoparticles; manipulating the size and surface charge of the LNPs to influence their biodistribution and reduce hepatic uptake—smaller, neutrally charged particles often exhibit prolonged circulation times and reduced liver accumulation; employing co-delivery strategies, where LNPs are combined with other agents that competitively inhibit hepatic uptake mechanisms, essentially saturating the liver’s ability to sequester the nanoparticles; or even leveraging alternative routes of administration, such as local injection directly into the affected tissue or region, to bypass the systemic circulation and minimise liver exposure altogether. Also, there are strategies like applying temporary stealth coatings with anchored two-armed polyethylene glycol (PEG) to the liver’s blood vessels, aiming to redirect nanomedicines to targeted organs [64]. The development of novel delivery strategies is crucial for realising the promise of RNA-based therapies beyond liver-specific applications.

In 2023, there were approximately 50,000 patients with severe hATTR. AHP affects fewer than 80,000 individuals, and PH1 affects no more than 30,000. These conditions affect fewer than 160,000 people. The frequency of high LDL-C levels according to [65] accounts for about 0.6% of the population, i.e., about 48 million people worldwide, but in practice, only a small proportion of these patients plan active treatment. Thus, while effective for the relatively small patient populations affected by these diseases, the approved drugs have limited socio-economic importance. Interest in them is mainly associated with the prospects of expanding the scope of RNA-containing drugs for the treatment of major diseases. However, RNA-based drugs for treating widespread diseases such as HIV, hepatitis C, various cancers, and CV diseases have yet to be approved.

The drug lademirsen for the therapy of Alport syndrome was approved for clinical trials in 2016. Having completed Phase 2 clinical trials long ago, the manufacturers appear to have abandoned further development of these drugs. Although publicly available reports on clinical trials of these drugs emphasise their high clinical efficacy and safety, it is likely that a substantial number of patients participating in the trials showed no signs of improvement. Furthermore, attempts to increase the dosages of the drugs to achieve a therapeutic effect sometimes resulted in severe consequences, including lethal cases. Currently, all RNA-based drugs approved for clinical use are designed to suppress gene expression in the liver. Delivery of short RNA to other organs, including kidneys (lademirsen) and vascular endothelium, remains ineffective. Sylentis S.A., a Spanish company, developed tivanisiran, which treats dry eye disease using local delivery of unmodified RNA. However, this approach proved ineffective.

We hypothesise that the current limitations stem from two key factors: inefficient delivery of RNA to target cells and a lack of specificity in the interaction of short RNAs with their intended cellular RNA targets. This is particularly relevant for miRNA mimetics and miRNA capturers, whose targets are not fully elucidated, leading to potentially unpredictable side effects.

### Next-Generation Delivery Strategies

Beyond LNPs and GalNAc conjugates, various other delivery methods are being explored to shuttle mRNA into cells. These include viral vectors like adeno-associated viruses (AAVs), which offer high transduction efficiency but face immunogenicity concerns. Exosomes, naturally secreted vesicles from cells, are being investigated for their inherent biocompatibility and ability to cross biological barriers. Other strategies include the use of inorganic nanoparticles, such as gold or silica nanoparticles, which can be modified with targeting ligands and associated with mRNA. Finally, physical methods, such as electroporation—an approach that uses electrical pulses to temporarily permeabilise cell membranes—and microinjection, which entails the direct injection of mRNA into cells, offer alternative delivery strategies. However, these methods are often restricted to in vitro experiments or local applications.

Polymer-based carriers are increasingly considered a promising alternative to LNPs for delivering mRNA. They offer potential solutions to some of the limitations inherent in LNP systems. However, ensuring adequate protection of the RNA from degradation by ribonucleases (RNases) remains a significant challenge during systemic administration. Novel polymeric RNA delivery systems must address the persistent vulnerability of RNA to breakdown, even when encapsulated within seemingly stable complexes [66].

We can hypothesise that improvements of RNA therapeutics delivery targeting can be achieved by applying new principles that have undergone limited testing in the clinic: the use of locked nucleic acids based on 2′–4′ constrained ethyl (cEt) nucleotides, which can self-penetrate myeloid-type cells without specialised delivery vehicles [67], and the use of exosomes enriched with PTGFRN and BASP1 receptors [68]. There may be some promise in abandoning the use of RNA interference in favour of antisense oligonucleotides containing locked nucleic acid (LNA-ASOs) that form abnormally strong duplexes with mRNA targets.

The potential of viral delivery of regulatory RNA using viral vectors, primarily adeno-associated virus (AAV), remains underutilised. However, this approach faces the challenge of reducing the nonspecific immune response to the virus or reducing the necessary RNA doses. Reducing the required RNA doses could be achieved by amplifying siRNA in vivo with the participation of RNA-dependent RNA polymerase, a process observed during RNA interference in invertebrates [69] and plants [70,71]. Alternatively, improving RNA delivery efficiency by minimising losses during transit through liposomes can be achieved by increasing the use of pH-reactive carriers such as amphoteric lipids and PBAE [72,73].

While many RNA therapeutics demonstrate durable effects, our understanding of their long-term impact on gene expression and cellular function remains limited. What are the potential risks of sustained target knockdown? Are there mechanisms of compensatory gene regulation that could diminish the therapeutic benefit over time? Longitudinal studies are needed to assess the durability and safety of RNA therapeutics over years and decades.

Although chemical modifications have reduced the immunogenicity of RNA therapeutics, the potential for unforeseen immune responses remains a concern, particularly with repeated dosing. What are the key molecular sensors that detect RNA and trigger immune activation? Can such immune activation be beneficial for treating viral infections and tumours? Can we develop strategies to further minimise immune stimulation while maintaining therapeutic efficacy? How does the immune status of the patient influence the safety and efficacy of RNA therapeutics?

Future research needs to prioritise innovative delivery systems. These systems should not only excel at targeting specific tissues, facilitating cellular uptake, and enabling escape from endosomes, but also protect the RNA molecule itself. Maintaining RNA integrity throughout the delivery process is crucial. Potential strategies include optimising the composition of LNPs to amplify targeting and intracellular delivery; engineering peptides designed to selectively bind to receptors; loading exosomes with RNA therapeutics and tailoring their surface to enhance targeting; and employing microfluidic devices for the more efficient production of LNPs.

## 4. Conclusions

In conclusion, RNA therapeutics hold immense promise for treating a range of diseases, particularly those with a genetic basis. While significant progress has been made with siRNA-based drugs targeting the liver, challenges remain in achieving efficient and targeted delivery to other tissues and minimising off-target effects. Future research should focus on developing novel delivery systems (e.g., exosomes, antibody conjugates), improving bioinformatics tools for target prediction, and rigorously validating target specificity using a combination of in vitro and in vivo assays. Furthermore, exploring the potential of other RNA therapeutic modalities, such as ASOs and mRNA therapies, will broaden the scope of this field. Overcoming these challenges is crucial to realising the full transformative potential of RNA therapeutics and developing safe, effective, and widely applicable treatments for a multitude of diseases.

## Figures and Tables

**Table 1:** A list of approved and investigational RNA-based drugs, organised by approval year (if applicable). Drugs utilising an LNP delivery system are indicated in blue. Approved drugs are indicated in green and not approved in orange.

Drug Name	Therapeutic Application	Molecular Target	RNA Type	Delivery System
Approved				
Patisiran/Onpattro/ALN-TTR02 (FDA, 2018)	Treatment of hATTR (familial amyloid polyneuropathy)	TTR	siRNA	100 nm LNP: cholesterol, DSPC, PEG2000-C-DMG, DLin-MC3 DMA
Givosiran/Givlaari (FDA, 2019)	Treatment of the AHP	ALAS1	siRNA	Conjugated with triantennary GalNAc
Inclisiran/Leqvio (EP, 2020)	Therapy of high LDL-C and an increased risk of premature atherosclerotic CV disease	PCSK9	siRNA	Conjugated with triantennary GalNAc
Lumasiran/Oxlumo (FDA, 2020)	Therapy of PH1	GO	siRNA	Conjugated with triantennary GalNAc
Vutrisiran/Amvuttra (FDA, 2022, 2025)	Treatment of hATTR (familial amyloid polyneuropathy)	TTR	siRNA	Conjugated with triantennary GalNAc
Nedosiran/Rivfloza (FDA, 2023)	Therapy for all types of primary hyperoxaluria	LDH	siRNA	Conjugated with triantennary GalNAc
Fitusiran/Qfitlia (FDA, 2025)	Therapy of haemophilia A/B	Antithrombin	siRNA	Conjugated with triantennary GalNAc
Not approved				
Cemdisiran	Therapy of IgAN, paroxysmal nocturnal haemoglobinuria, myasthenia gravis, atypical haemolytic uremic syndrome	C5	siRNA	Conjugated with triantennary GalNAc
Cosdosiran	Therapeutic for NAION and primary angle closure glaucoma	Caspase 2	siRNA	No
Lademirsen/RG012	Treatment of Alport syndrome	miR-21	ss miR-capturer	No data
MRG110	Wound healing and preventing HF	miR-92	ss miR-capturer	No data
Remlarsen/MRG201	Prevention of fibrous and keloid scar formation	miR-29 (collagens)	ds miR mimetic	No
Teprasiran	Therapy of DGF	p53 anti-oncogene	siRNA	No
Tivanisiran	Treatment of dry eye disease	TRPV1 receptor	siRNA	No

**Table 2:** Administration route, efficacy, and side effects for approved and investigational RNA-based drugs (alphabetically). Approved drugs are indicated in green and not approved in orange.

Drug Name	Administration Route	Efficacy	Side Effects	Reference
Approved				
Fitusiran/Qfitlia (FDA, 2025)	Subcutaneous	Reduction in bleeding rate. Maintained effective bleed protection over a 6-year period. Improved health-related quality of life	Viral or bacterial infection, nasopharyngitis, abnormal blood clotting, gallbladder disease symptoms, elevated transaminases	NCT03417102 NCT03417245 NCT05662319
Givosiran/Givlaari (FDA, 2019)	Subcutaneous	Reduction in urinary aminolevulinic acid (ALA) and porphobilinogen (PBG). Reduction in annualised attack rate of acute hepatic porphyria (AHP) attacks vs. placebo	Nausea, injection site reactions, rash, fatigue, elevated transaminases, renal toxicity	NCT03338816
Inclisiran/Leqvio (EP, 2020)	Subcutaneous	Reduction in LDL-C from baseline, on top of statin therapy. Effects sustained with biannual dosing	Injection site reaction, arthralgia, fatigue, myalgia	NCT03397121, NCT03399370, NCT03400800
Lumasiran/Oxlumo (FDA, 2020)	Subcutaneous	Reduction in urinary oxalate levels from baseline in adults	Injection site reactions, abdominal pain	NCT03681184 NCT03905694
Nedosiran/Rivfloza (FDA, 2023)	Subcutaneous	Reduction in oxalate production in children aged ≥ 9 years and adults with PH1	Injection site reactions, severe and fluctuating tachycardia	NCT04555486 NCT05001269 NCT03847909
Patisiran/Onpattro/ALN-TTR02 (FDA, 2018)	Intravenous	Improved mNIS+7 neuropathy score vs. placebo at 18 months. Improved quality of life, mobility, and nutritional status	Infusion-related reactions, upper respiratory tract infections	NCT01960348
Vutrisiran/Amvuttra (FDA, 2022, 2025)	Subcutaneous	Reduction in urgent HF visits, risk of CV events, hospitalisations, and mortality through 36 months. Improved mNIS+7 neuropathy score vs. placebo at 9 months	Injection site reactions, pain in the limbs and joints, shortness of breath, and low vitamin A levels	NCT05635045 NCT03759379
Not approved				
Cemdisiran	Subcutaneous	Substantial reduction in proteinuria in patients with IgA nephropathy and PNH. Used with complement inhibitor pozelimab for PNH	Headache, cough, fatigue, nausea	NCT04888507 NCT03841448 (Phase 2)
Cosdosiran	Directly into the eye	The Phase 3 trial did not meet the primary endpoint	Intraocular inflammation, increased intraocular pressure, conjunctival hyperaemia	NCT03913130 (Phase 3)
Fitusiran	Subcutaneous	Reduction in annualised bleeding rate (ABR) in haemophilia A and B patients (with/without inhibitors)	Injection site reactions, transaminase elevations, thrombotic events	NCT03417102, NCT03417245
Lademirsen/RG012	Subcutaneous	The Phase 2 study was completed but did not show significant efficacy on the primary endpoint	-	NCT02855268
MRG110	Intradermal injection	Targets miR-92 to promote angiogenesis and healing, as shown in Phase 2	-	NCT03603431 (Phase 1)
Nedosiran/Rivfloza	Subcutaneous	Reduction in urinary oxalate from baseline in PH1 patients. A significant proportion reached normal/near-normal levels	Injection site reactions, abdominal pain, fatigue, headache	NCT03847909
Remlarsen/MRG201	Intradermal injection	Mimics miR-29 to suppress fibrosis	Injection site reactions (discomfort, discolouration, swelling)	NCT03601052
Teprasiran	Single intravenous infusion	The Phase 3 trial results do not meet primary endpoint criteria	A higher incidence of hypotension was observed in the teprasiran group compared to placebo	NCT03510897
Tivanisiran	Ophthalmic eye drops	Topical siRNA targeting the nerve growth factor receptor (NGFR)	Instillation site pain, irritation, blurred vision	NCT03108664
Vutrisiran/Amvuttra	Intravenous	Reversing disease progression at 9 months	Injection site reactions	NCT03759379

DL-C = low-density lipoprotein cholesterol; mNIS+7 = modified Neuropathy Impairment Score +7; PH1 = primary hyperoxaluria type 1; PNH = paroxysmal nocturnal haemoglobinuria; IgAN = Immunoglobulin A Nephropathy.
